# Outcomes of deep venous thrombosis management and associated factors among patients in tertiary hospitals in Addis Ababa, Ethiopia: a multicenter retrospective cohort study

**DOI:** 10.1186/s12959-024-00627-2

**Published:** 2024-07-12

**Authors:** Seble Birhane, Melak Gedamu Beyene, Fishatsion Tadesse, Assefa Mulu Baye

**Affiliations:** 1 Department of Pharmacology and Clinical Pharmacy, School of Pharmacy, College of Health Sciences, Addis Ababa University, P.O. Box 1176, Addis Ababa, Ethiopia; 2 Department of Internal Medicine, School of Medicine, College of Health Sciences, Addis Ababa University, Addis Ababa, Ethiopia

**Keywords:** Deep venous thrombosis, Recurrence, Bleeding, Addis Ababa

## Abstract

**Background:**

Pulmonary embolism (PE) and deep venous thrombosis (DVT) are the two most important manifestations of venous thromboembolism (VTE). DVT remains a significant condition since associated morbidity is significant and has elevated healthcare-related costs.

**Methods:**

A retrospective cohort study was conducted among DVT patients admitted to Tikur Anbessa Specialized Hospital, Zewditu Memorial Hospital and St. Paul’s Hospital Millennium Medical College on follow-up from July 1, 2017, to July 01, 2020. Data on sociodemographic characteristics, types of DVT, laboratory findings, medications, risk factors of DVT, complications and outcomes of DVT were collected. The data were analyzed using SPSS version 25. Multivariate logistic regression analysis was conducted to determine predictors of DVT recurrence and major bleeding. A P value < 0.05 was considered to identify significant predictors.

**Results:**

The mean age of the participants was 45.2 years, with SD of 15.36. The major causes of DVT included immobilization (29.9%), previous surgery (27.5%) and cancer (21.1%). The DVT recurrence rate was 22.5%. Nine (2.2%) of the participants died, and 19.9% developed complications. Bilateral DVT (Adjusted odds ratio (AOR) = 2.8, 95% Confidence interval (CI) = 1.14, 6.66), obesity (AOR = 3.3, 95% CI = 1.15, 9.59), hypertension (AOR = 6.5, 95% CI = 2.90, 14.70) and retroviral infection (AOR = 6.3, 95% CI = 2.34, 16.94) were predictors of recurrent DVT. Nineteen (4.7%) patients had major bleeding, and patients with bilateral DVT, active cancer and terminal age had an increased risk of major bleeding.

**Conclusions:**

The overall DVT recurrence rate was alarmingly high and further complicated by PE, post thrombotic syndrome and chronic vein insufficiency, resulting in a 2.2% death rate. Major bleeding after DVT and PE remained high. Close monitoring should be performed for patients with advanced age, active cancer, bilateral DVT, retroviral infection, obesity and hypertension to prevent the recurrence of DVT and major bleeding.

## Introduction

Venous thromboembolism (VTE), including deep venous thrombosis (DVT) and/or pulmonary embolism (PE), is a common, severe, potentially fatal but manageable acute condition. Approximately 0.1% of the global population each year develop DVT [1]. DVT is a complex multifactorial disease and an important cause of preventable mortality and morbidity [2]. The incidence of DVT increases sharply after approximately 45 years of age and is slightly greater in men than in women of advanced age. Major risk factors for thrombosis, other than age, include exogenous factors such as surgery, hospitalization, immobility, trauma, pregnancy, and puerperium and hormone use and endogenous factors such as cancer, obesity, and inherited and acquired disorders of hypercoagulation [3].

Globally, DVT remains a significant condition since associated morbidity is significant and has elevated healthcare-related costs [4]. DVT and associated complications are common in hospital settings, but they are also becoming more common among outpatients. The incidence of VTE was 5.5% among hospitalized patients in a study conducted in Addis Ababa [5]. A study from Addis Ababa and Jimma reported that the recurrence rate of DVT was 26.4%, which is higher than most previously published data [6]. A study conducted in Canada revealed that the rates of recurrent VTE after DVT and PE remain unacceptably high, where one-fifth of cases experienced recurrent VTE [7]. According to a study in the US, DVT contributes to the deaths of 33% of those affected [8]. VTE is a major health problem in Europe, with more than one million VTE events or deaths per year in the six countries examined. Almost three-quarters of all VTE-related deaths were reported from hospital-acquired VTE [9].

According to the Centers for Disease Control and Prevention (CDC), one-third of patients diagnosed with DVT/PE experience recurrence within 10 years, and up to 50% develop post thrombotic syndrome (PTS) [10]. The frequency of recurrent DVT is increasing, making early identification and treatment of risk factors that predispose patients to recurrence critical.

The aim of treatment for DVT is to reduce morbidity and mortality. This is achieved by optimal therapy with anticoagulants to prevent thrombus extension and embolization, thus preventing recurrence and death, which exposes patients to an inherent increased risk of bleeding [11]. In a study from Canadan, among patients with DVT, 12.8% developed major bleeding during the follow-up period [7].

There is a dearth of information available in Ethiopia on the outcomes of DVT treatment and the risk factors for its recurrence. Most prior studies that examined the recurrence rate of DVT in Ethiopia had limited sample sizes and relied on nonmulticenter data, and most disregarded secondary outcomes such as major bleeding. Therefore, this study was conducted to evaluate treatment outcomes and associated factors among DVT patients receiving anticoagulation therapy at three tertiary care hospitals, namely, Tikur Anbessa Specialized Hospital (TASH), St. Paulos Hospital Millennium Medical College (SPHMMC) and Zewuditu Memorial Hospital (ZMH), in Addis Ababa.

## Methods and materials

### Study design, area and period

A retrospective cohort study was conducted among DVT patients at SPHMMC, ZMH and TASH. We chose these hospitals because they are referral hospitals which allows us to find diverse participants. During the data collection period, there were approximately 720 patients under regular follow-up at the clinics of the hospitals, with an average of 30 patients seen at each clinic daily. The data were collected from October 1, 2020 to March 31, 2021.

### Population

DVT patients at TASH, SPHMMC and ZMH who were on follow-up between July 1, 2017, and July 1, 2020, who were aged 18 years and older, and who were on anticoagulants for at least 3 months or more were included. DVT patients who had incomplete data or medical records were excluded from the study.

### Sample size determination

A total of 408 study participants were included using a single proportion population formula and were proportionally allocated to TASH, SPHMMC or ZMH. Systematic random sampling techniques were employed to select participants.

### Data collection tools and procedures

The data collection tool was developed by using different studies [12–14]. The data collected included sociodemographic characteristics, types of DVT, laboratory findings, a list of all medications, risk factors and clinical data, complications and outcomes of DVT. Information about the category of bleeding that occurred during the treatment period was evaluated based on the International Society on Thrombosis and Hemostasias [14]. All the data were collected from patient medical records.

The data were collected by two trained data collectors, one nurse and one pharmacist trained on the objective of the study and the contents of the questionnaire.

The validity of the study was checked by a pretest performed on 5% of the study population at St. Peter Specialized Hospital with similar characteristics to ensure the agreement of the data abstraction format with the needs of the study. Modifications were mainly made to the variables used to measure DVT treatment outcomes and were corrected and modified into the final version of the data collection tool. Continuous supervision was provided by the principal investigator to ensure the completeness and consistency of the collected data. All collected data were examined for completeness and consistency during data management, storage and analysis.

### Data Analysis

The data were collected and entered into Epi info 4.6.0.6 and analyzed using Statistical Package for the Social Sciences (SPSS) version 25. Descriptive statistics such as frequency, percentage, mean, and standard deviation (SD) were used to describe demographic and clinical features, as well as the outcomes. Logistic regression models were used to determine whether the sociodemographic data, laboratory findings and comorbid conditions were related to outcomes such as recurrent DVT and major bleeding. Candidate variables possibly associated with the outcome variables (P value < 0.25) after bivariate analysis were included in the multivariable logistic regression model. A P value < 0.05 was considered a significant predictor of the outcome.

### Outcome measures

The primary outcomes for the study were recurrence, major bleeding and death, whereas the secondary outcomes were factors for recurrent DVT and major bleeding.

### Definition of terms

#### DVT recurrence

was defined as an objectively verified hospital discharge diagnosis of DVT, a fatal complication of DVT confirmed by ultrasonography, or a site of thrombosis that was either previously uninvolved or had interval documentation of incident DVT [14].

#### Major bleeding

included fatal bleeding; retroperitoneal, intracranial, or intraocular bleeding; bleeding that causes hemodynamic compromise requiring specific treatment; bleeding that requires intervention (surgical or endoscopic) or decompression of a closed space to stop or control the event; clinically overt bleeding, requiring any transfusion of one unit or more of packed red blood cells or whole blood; and clinically overt bleeding, causing a decrease in hemoglobin of 3 g/dL or more (or, if the hemoglobin level is not available, a decrease in hematocrit of 10% or greater) [15].

#### Complication

Complications from deep vein thrombosis can be very serious. It includes pulmonary embolism (PE), chronic venous insufficiency, and post thrombotic syndrome [16].

## Results

### Sociodemographic and clinical characteristics

The study sample consisted of 408 men and women from the three hospitals during the three-year study period and included patients who had taken anticoagulant medication for at least 3 months. The mean age and SD of the study participants were 45.2 and 15.36 years, respectively. As shown in Table 1, approximately one-third (36.5%) of the participants were in the ≥ 51 years age group. More than half (58.8%) of the participants were female.

The majority of DVT patients reported had lower limb thrombosis (95.3%), followed by unilateral (78.7%) and proximal DVT (40.4%). Regarding the presence of signs and symptoms, more than three-fourths of the studied patients had pitting edema, and all the studied patients experienced pain and swelling. Among the risk factors for DVT, immobilization was associated with 29.9% of the patients, surgery was associated with 27.5% of the patients, and the other risk factors were summarized. The most commonly reported comorbid diseases were hypertension (30.4%), diabetes mellitus (11.8%), retroviral infection (11.4%), and malignant diseases (62, 15%). Breast cancer was the most common cancer, accounting for 29% of the malignant cases, followed by ovarian, cervical, rectal and liver cancers. 18% of the participants had a prior history of surgery (Table 1).

**Table 1:** Sociodemographic characteristics and clinical profiles of study participants with DVT at selected hospitals in Addis Ababa, July 1, 2017 to July 1, 2020

Characteristics		Frequency	Percent
Age (years)	18–35	116	28.4
	36–50	143	35.1
	≥ 51	149	36.5
Sex	Male	168	41.2
	Female	240	58.8
Sites of DVT	Lower limb	389	95.3
	Upper limb	6	1.5
	Both lower and upper limbs	13	3.2
DVT types	Proximal	165	40.4
	Distal	11	2.7
	Both	232	56.9
DVT involved limb	Unilateral	321	78.7
	Bilateral	87	21.3
Sign and symptoms at presentation	Pain	408	100.0
	Swelling	408	100.0
	Pitting edema	318	77.9
	Skin discoloration	233	57.1
	Local tenderness	219	53.7
Risk factors for DVT	Recent immobilization	122	29.9
	Previous surgery	112	27.5
	Active cancer	86	21.1
	Previous VTE	84	20.6
	Presence of infection	80	19.6
	Pregnancy	64	15.7
	Obesity	54	13.2
	Age > 75	33	8.1
	Heart failure	23	5.6
	Stroke	18	4.4
	Prior history of trauma	17	4.2
	Genetics	9	2.2
	Unprovoked	94	23.0
Comorbidity		204	50.0
Hypertension		124	30.4
Prior history of surgery		74	18.1
Types of surgery	Abdominal	41	55.4
	Orthopedic	17	23.1
	Brain	6	8.1
	ENT	5	6.8
	Cardiac	3	4.1
	Breast	2	2.7
Cancer		62	15
Types of cancers	Breast	18	29.0
	Ovarian	17	27.4
	Cervical	12	19.4
	Rectal	10	16.1
	Other *	5	8.0
RVI		59	14.4
Diabetes mellitus		48	11.8
HF		21	5.1
Dyslipidemia		13	3.2
Stroke		12	2.9
CLD		11	2.6
AF		3	0.7

*Lung, colorectal and liver cancer; AF, atrial fibrillation; CLD, chronic liver disease; DVT, deep vein thrombosis; ENT, eye, nose and throat; HF, heart failure; RVI, retroviral infection

The majority of the study participants were taking warfarin (79.4%), along with either unfractionated heparin (80.8%) or enoxaparin (19.1%), and the rest were taking rivaroxaban (20.6%). All of the study participants were on anticoagulants. Among a total of 408 DVT patients, 204 had comorbid conditions and were taking concurrent medications. The majority of these patients were taking antibiotics, followed by cardiovascular, vitamin K, and antidiabetic drugs, as shown in Table 2.

**Table 2:** Anticoagulants and other medications used for the management of DVT and comorbidities at selected hospitals in Addis Ababa, July 01, 2017 - July 01, 2020

Anticoagulants medications	Frequency	Percent
Warfarin	324	79.4
Heparin	262	80.8
Enoxaparin	62	19.1
Rivaroxaban	84	20.6
Antibiotics	55	13.5
Calcium channel blockers	43	10.5
ACE inhibitors	33	8.1
Vitamin K	30	7.4
Statins	29	7.1
Beta blockers	24	5.9
Anti-diabetics	23	5.6

ACEI: Angiotensin Converting Enzyme Inhibitor

The following table presents the bleeding status of the DVT patients. The results showed that 42 (10.0%) patients had a history of bleeding, and 19 (4.7%) had major bleeding (Table 3).

**Table 3:** Bleeding status of the study participants according to the International Society on Thrombosis and Hemostasias guidelines at selected hospitals in Addis Ababa, July 01, 2017 - July 01, 2020

Variables		Frequency	Percent
Bleeding during follow up		42	10.0
Patients with major bleeding		19	4.7
Subjective finding of bleeding (*n* = 28)	Nose bleeding	11	39.3
	Active vaginal bleed	6	21.4
	GI bleeding	4	14.3
	Gum bleeding	3	10.7
	Hematuria	3	10.7
	Urethral bleeding	1	3.6

GI: Gastrointestinal tract

According to our study findings, the incidence of recurrent DVT was 22.5%, and the majority of patients experienced two recurrences of DVT (20.1%). After treatment with anticoagulants, 17.9% of patients were completely resolved, 10% worsened, and 2.2% died. Approximately one-fifth (19.9%) of the study participants developed complications, and PE accounted for 44.4%, followed by PTS and chronic vein insufficiency (Table 4).

**Table 4:** DVT treatment outcomes at selected hospitals in Addis Ababa, July 01, 2017 - July 01, 2020

Variables		Frequency	Percent
Patient develops recurrent DVT		92	22.5
Number of recurrent DVT	Two	82	20.1
	Three	8	2.0
	Five	2	0.5
Time of recurrence (year)	< 1	21	22.8
	1–3	35	38
	3–5	20	21.7
	> 5	16	17.4
Current status of DVT	Improved	210	51.5
	No change	75	18.4
	Complete resolution	73	17.9
	Prophylaxis after complete resolution (*n* = 73)	20	27.4
	Worsened	41	10.0
	Death	9	2.2
Cause of death	RF secondary to massive PE	4	44.4
	RF secondary to acute PE	2	22.2
	Others*	3	33.3
Developed complication		81	19.9
Types of complication	PE	36	44.4
	PTS	31	38.3
	Chronic venous insufficiency	14	17.3

*Bleeding secondary to supra-INR, RF secondary to PE + septic shock and UGIB & hypoxia secondary to CLD; PE: pulmonary embolism, PTS: post thrombotic syndrome, RF: respiratory failure, UGIB: upper gastrointestinal bleeding, DVT: deep venous thrombosis

### Predictors of recurrent DVT

The results of the multivariate logistic regression analysis showed that bilateral DVT, obesity, hypertension and the RVI were significantly associated with DVT recurrence. Accordingly, participants with bilateral DVT had a 2.8-fold greater likelihood of recurrence than did participants with unilateral DVT (AOR = 2.8, 95% CI = 1.14, 6.66), and obese participants had a 3.3-fold greater likelihood of recurrence than did participants with a normal body mass index (AOR = 3.3, 95% CI = 1.15, 9.59). Participants with hypertension had a 6.5-fold greater recurrence of DVT than did nonhypertensive patients (AOR = 6.5, 95% CI = 2.90, 14.70), while RVI patients had a 6.3-fold greater recurrence of DVT than did their counterparts (AOR = 6.3, 95% CI = 2.34, 16.94) (Table 5).

**Table 5:** Multivariate binary logistic regression analysis of predictor factors for DVT recurrence among study participants at selected hospitals in Addis Ababa, July 01, 2017 - July 01, 2020

Variables		Recurrence DVT		COR (95%CI)	AOR (95% CI)	P value
		Yes (%)	No (%)			
Age category	18–35	15(8.6)	101(91.4)	Reference		
	36–50	41(28.7)	102(71.3)	2.7(1.41, 5.19)	1.5(0.59, 3.84)	0.383
	>=51	36(24.2)	113(75.8)	2.1(1.11, 4.15)	0.79(0.29,2.17)	0.652
Gender	Male	25(14.9)	143(85.1)	Reference		
	Female	67(28.0)	173(72.0)	2.2(1.33, 3.69)	0.59(0.28, 1.25)	0.171
Types of DVT	Unilateral	43(13.6)	278(86.6)	Reference		
	Bilateral	49(56.3)	38(43.7)	8.3(4.89, 14.19)	2.8(1.14, 6.66)	0.024*
Local tenderness	Yes	56(25.6)	163(74.4)	1.5(0.91, 2.34)	0.64(0.32, 1.29)	0.218
	No	36(19.1)	153(80.9)	Reference		
Presence of infection	Yes	48(60.0)	32(40.0)	9.7(5.59, 16.76)	1.2(0.37, 2.92)	0.944
	No	44(13.4)	284(86.6)	Reference		
Obesity	Yes	37(68.5)	17(31.5)	11.8(6.22, 22.49)	3.3(1.15, 9.59)	0.027*
	No	55(15.6)	299(84.4)	Reference		
Previous surgery	Yes	46(41.1)	66(58.9)	3.8(2.32, 6.19)	0.68(0.27, 1.68)	0.410
	No	46(15.6)	250(84.4)	Reference		
Recent immobilization	Yes	48(30.4)	74(60.6)	3.6(2.19, 5.79)	0.94(0.42, 2.15)	0.893
	No	44(16.4)	242(84.6)	Reference		
Hypertension	Yes	65(52.4)	59(47.6)	10.5(6.17, 17.82)	6.5(2.90, 14.70)	0.000*
	No	27(9.5)	257(90.5)	Reference		
Retroviral infection	Yes	46(79.3)	12(20.7)	23.3(11.69,46.44)	6.3(2.34, 16.94)	0.000*
	No	46(13.2)	303(86.8)	Reference		
Heparin use	Yes	55(20.0)	220(80.0)	Reference		
	No	37(28.4)	96(71.6)	1.5(0.95, 2.49)	0.70(0.34, 1.46)	0.345

*p value < 0.05; DVT: deep venous thrombosis, INR: international normalized ratio, COR: crude odds ratio, AOR: adjusted odds ratio, CI: confidence interval

### Predictors of major bleeding

In addition, the strength of the association between the independent variable and major bleeding was alsomeasured using a 95% confidence interval and odds ratio. Accordingly, DVT type, the presence of active cancer and age older than 75 years were associated with major bleeding according to multivariable logistic regression analysis.

Patients with bilateral DVT had a 3.9-fold increase in major bleeding compared with patients with unilateral DVT (AOR = 3.9, 95% CI = 1.6, 9.7), and participants with active cancer disease had a 6.5-fold increase in major bleeding compared with those with no active cancer (AOR = 6.5, 95% = 2.9, 14.75). Participants aged >75 years had a 6.8-fold greater incidence of major bleeding than did those aged younger than 75 years (AOR = 6.8, 95% CI = 2.03, 22.33) (Table 6).

**Table 6:** Multivariate binary logistic regression analysis of predictors associated with major bleeding at selected hospitals in Addis Ababa, July 01, 2017 - July 01, 2020

Variables		Major bleeding		COR (95%CI)	AOR (95% CI)	P value
		Yes (%)	No (%)			
Gender	Male	14(8.3%)	154(91.7)	Reference		
	Female	28(11.7%)	212(88.3)	1.5(0.74, 2.85)	0.821(0.35, 1.94)	0.653
Types of DVT	Unilateral	16(4.4%)	305(95.6)	Reference		
	Bilateral	26(29.9%)	61(70.1)	8.1(4.11, 16.05)	3.94(1.6, 9.701)	0.003*
Pitting edema	Yes	35(11%)	283(89.0)	1.5(0.63, 3.42)	0.74(0.26, 2.13)	0.573
	No	7(7.8%)	83(92.2)	Reference		
Active cancer	Yes	28(32.6%)	58(67.4)	10.6(5.27, 21.39)	6.54(2.90, 14.75)	0.000*
	No	14(4.3%)	308(95.7)	Reference		
Diabetes mellitus	Yes	18(47.5%)	30(62.5)	8.4(4.10, 17.19)	1.4(0.42, 4.70)	0.577
	No	24(6.7%)	336(93.3)	Reference		
Age > 75 years	Yes	19(57.6%)	14(42.4)	20.7(9.25, 46.64)	6.8(2.03, 22.93)	0.002*
	No	23(6.1%)	352(93.9)	Reference		

*p value < 0.05, DVT: deep venous thrombosis, COR: crude odds ratio, AOR: adjusted odds ratio, CI: confidence interval

## Discussion

The current study investigated the incidence and predictors of recurrent DVT in three selected tertiary hospitals in Addis Ababa, Ethiopia. A higher rate of recurrent DVT was reported, accounting for 22.5%. Nine participants (2.2%) died, and 19.9% of patients developed complications. PE accounted for 44.4% of these complications, followed by PTS and chronic vein insufficiency. Forty-two participants (10%) experienced bleeding episodes, and 4.7% of the participants experienced major bleeding.

Among the signs and symptoms, pain and general swelling manifested in all patients. Similar findings were reported in Ethiopia and Hong Kong [6, 17]. Unilateral limb involvement was observed in 78.7% of the patients. A higher percentage of unilateral involvement was also previously reported in Addis Ababa [18]. Our study also showed that the majority of the patients (95.3%) had DVT in their lower limbs, which is in line with a study performed in Zambia [19] and Hong Kong [17].

Our findings showed that the most common causes of DVT were a longer hospital stay (immobilization) (29.9%), previous surgery (27.5%), active cancer (21.1%), previous VTE (20.6%), acute infection (19.6%), pregnancy (15.7%), heart failure (5.6%), prior history of trauma (4.2%), presumed genetic disorders (2.2%) and age ≥ 75 years (8.1%), while the remaining 23% had unprovoked DVT. According to a previous study performed in Addis Ababa [18], the most commonly noted causes of DVT were malignancy of any kind (30.9%) and prolonged immobilization (19.8%). The difference may be that the current study was a multicenter study. Likewise, a study from America [1] reported that cancer was also a common comorbid condition, affecting 36.4% of all patients, while recent immobility and/or injury were found in 41.9% of the cohort and 22.8% of patients with the event classified as unprovoked. The finding of unprovoked risks was similar to our findings. In contrast, a study in Nigeria [20] showed that only 2% were unprovoked. This variation might be due to the smaller sample size used in the Nigerian study.

The incidence of recurrent DVT was 22.5%, and the majority of patients experienced two recurrences of DVT (20.1%). This finding was comparable to those of previous studies performed in Ethiopia [6] and the USA [21], which reported DVT recurrence rates of 26.4% and 26.1%, respectively. However, a lower incidence of recurrent DVT has been reported elsewhere [22]. The variation might be attributed to methodological differences and patient care disparities in various settings.

The results of the multivariate logistic regression analysis showed that bilateral DVT, obesity, hypertension and RVI were significantly associated with DVT recurrence. In our study, the odds of recurrent DVT were 6.5 times greater among patients with hypertension than among patients without hypertension. Similarly, hypertension was a predictor of recurrent DVT in a meta-analysis [23]. This could be explained by elevated blood pressure causing damage to venous valves, resulting in obstruction of blood vessels [24], and hypertension can also be associated with hypercoagulability [25].

In addition, the odds of developing recurrent DVT among people living with HIV (PLWH) were 6.3 times greater than those among their counterparts. A similar finding was also reported in the Netherlands, where the risk of recurrent VTE was elevated in PLWH compared to controls [26]. HIV-associated immunosuppression, opportunistic infections, malignancies and coagulopathy abnormalities contribute to an increased risk of recurrent DVT [27].

Our findings revealed that bilateral DVT was associated with a 2.8-fold increase in recurrent DVT. Similarly, a report from Qatar revealed that bilateral DVT predisposes patients to recurrent DVT [28].

The risk of recurrent DVT among obese patients was 3.3 times greater than that among nonobese patients. Similar findings were reported elsewhere [23, 29]. Although the pathophysiologic mechanisms underlying the contribution of obesity to recurrent DVT have not been fully explained, several speculated factors might play a role. Obesity may be associated with a prothrombotic state characterized by increased levels of procoagulant factors and decreased levels of anticoagulant factors [30].

Our study revealed that 9 (2.2%) of the participants died during the study period. The most common complication causing death was PE. This was comparable to that observed in the Worcester Venous Thromboembolism Study [7], where patients who presented with PE had a significantly greater overall death rate than those who presented with isolated DVT. In addition, Labropoulos et al. reported an association between PE and death [21].

Our data showed that 19.9% of patients developed complications, 44.4% of whom developed PE, 38.3% of whom developed PTS, and 17.3% of whom experienced chronic vein insufficiency. The CDC reported that 33–50% of people who have experienced DVT will experience long-term complications (post thrombotic syndrome), which include swelling, pain, discoloration, and scaling in the affected limb [31]. A study from Sweden showed that 26% of the study population developed PTS, 12% recurrent DVT, and 9% PE [32].

Our findings revealed that 4.7% of patients experienced major bleeding. Similar results were reported for Sweden (4.0%) [33] and Denmark (6.6%) [34]. The type of DVT (bilateral DVT), presence of active cancer and age above 75 years were associated with major bleeding. The likelihood of developing major bleeding among patients with bilateral DVT was 3.9 times greater than that among patients with unilateral DVT. This finding could be partly explained by the fact that bilateral DVT can cause major bleeding due to the extensive clot burden and the disruption of blood vessels [35], and intensive anticoagulation therapy further increases the risk of bleeding [36]. Participants with active cancer had a 6.5-fold increase in major bleeding. This finding is in line with a study from Japan [37]. This is explained by the complex nature of the pathophysiology of cancer and associated chemotherapy-induced bleeding [38]. Participants aged more than 75 years had a 6.8-fold increased risk of major bleeding. This was supported by another study performed in Italy [39].

Some investigations, including ours, have indicated that an age greater than 75 years is related to an increased risk of bleeding; hence, this parameter has been incorporated in the calculation of bleeding risk scores [39, 40]. Similar findings were reported elsewhere [41, 42], indicating that old age is a contributing factor to the risk of bleeding. This might be due to risk factors such as cancer, stroke, heart failure, immobilization, and a history of VTE, which are more common in the elderly population and contribute to a greater incidence of bleeding events [33].

In resource limited settings, the management and follow-up of DVT present unique challenges. Initial management typically involves intensive anticoagulant therapy, such as low molecular weight heparin or direct-acting oral anticoagulants, although access to these medications is limited. Follow-up care often relies on outpatient clinics, where patients may face barriers to regular attendance due to distance, transportation costs, or competing socioeconomic interests. These challenges contribute to suboptimal adherence to treatment and monitoring protocols, increasing the risk of recurrence and complications. Moreover, lack of specialty care facilities and trained health professionals may hinder the diagnosis and management of DVT complications, such as bleeding events. The implications of these systemic challenges are significant, potentially exacerbating the burden of recurrent and complications of DVT. Efforts to address these issues should prioritize improving access to essential medications, enhancing healthcare infrastructure, and support patient adherence and follow-up care.

This research employed a larger sample size in multiple study settings than did previous studies in Ethiopia. We aimed to incorporate key outcomes of DVT, including recurrence, mortality, complications, major bleeding, and predictive factors for recurrence and major bleeding, which have not been thoroughly examined in prior studies. However, our study is not without limitations. Since it is a retrospective study, it is predisposed to recall bias. We encountered instances of incomplete recording by providers or insufficient information regarding relevant findings, such as subjective bleeding assessments being unavailable in many cases. Additionally, most of the data associated with the INR were not recorded. Despite these limitations, our study provides valuable insights and offers important recommendations for future research and clinical practice.

## Conclusions

In the current study, the overall DVT recurrence rate was 22.5%, which was complicated by PE, PTS and chronic vein insufficiency, resulting in 2.2% death. Major bleeding rates after DVT and PE remain high. Efforts are needed to identify patients who are most at risk for VTE complications, and developing better anticoagulation strategies that are suitable for long-term use is crucial for better patient treatment outcomes. Close monitoring should be performed for patients with advanced age, active cancer, bilateral DVT, RVI, obesity and hypertension to prevent the recurrence of DVT and major bleeding.

## Data Availability

No datasets were generated or analysed during the current study.

## Abbreviations

DOAC: Direct oral anticoagulant
DVT: Deep vein thrombosis
INR: International normalized ratio
LMWH: Low-molecular-weight heparin
NOAC: Nonvitamin K antagonist oral anticoagulants
PE: Pulmonary embolism
PT: Prothrombin time
PTS: Post thrombotic syndrome
RVI: Retroviral infection
VTE: Venous thromboembolism
VKA: Vitamin K-Antagonist
