# Correction: Outcomes of deep venous thrombosis management and associated factors among patients in tertiary hospitals in Addis Ababa, Ethiopia: a multicenter retrospective cohort study

**DOI:** 10.1186/s12959-024-00639-y

**Published:** 2024-07-31

**Authors:** Seble Birhane, Melak Gedamu Beyene, Fishatsion Tadesse, Assefa Mulu Baye

**Affiliations:** 1 Department of Pharmacology and Clinical Pharmacy, School of Pharmacy, College of Health Sciences, Addis Ababa University, P.O. Box 1176, Addis Ababa, Ethiopia; 2 Department of Internal Medicine, School of Medicine, College of Health Sciences, Addis Ababa University, Addis Ababa, Ethiopia

**Correction: Thrombosis J 22**,** 62 (2024)**

10.1186/s12959-024-00627-2

Following publication of the original article, it was observed that a comment made by the authors during the proofing stage was included in the final published version.

The comment read as **“Predictors of recurrent DVT and major bleeding” shall be “Predictors of recurrent DVT””** and was located at the end of the first paragraph of the Introduction.

The original article has been corrected.

The publisher would like to apologize for any inconvenience this may have caused.

